# S183. ABNORMALITIES OF FRONTO-SUBCORTICAL PATHWAYS IN SCHIZOPHRENIA AND THE DIFFERENTIAL IMPACTS OF ANTIPSYCHOTIC TREATMENT: A DTI-BASED TRACTOGRAPHY STUDY

**DOI:** 10.1093/schbul/sby018.970

**Published:** 2018-04-01

**Authors:** Anaïs Vandevelde, Elise Leroux, Maxime Tréhout, Sonia Dollfus

**Affiliations:** Normandie University

## Abstract

**Background:**

The fronto-striato-thalamic circuitry is a key network associated with several symptoms observed in patients with schizophrenia (SZPs). In this study, we use diffusion tensor imaging (DTI) to investigate the integrity of white matter (WM) pathways involved in this network in SZPs relative to healthy controls (HCs). We also evaluate the differential impact of chronic exposure to clozapine as well as other atypical and typical antipsychotics on fasciculi integrity in this network in schizophrenia.

**Methods:**

63 HCs and 41 SZPs were included in this study. Of the SZPs, 16 were treated with clozapine (SZPsC), 17 with atypical antipsychotics (SZPsA), and 8 with typical antipsychotics (SZPsT). We reconstructed three tracts belonging to the fronto-striato-thalamic network in the left hemisphere using tractography: one fronto-subcortical tract (FSC), one prefronto-subcortical tract (PFSC), and one prefronto-frontal tract (PFF). Diffusion parameters were individually extracted in each tract.

**Results:**

SZPs exhibited lower integrity in both the FSC and PFSC relative to HCs, and SZPsT patients showed altered integrity compared to SZPsC patients. There were no WM integrity differences in the PFF between SZP groups or between SZPs and HCs.

**Discussion:**

These results suggest that SZPs exhibit structural connectivity abnormalites in the prefronto-fronto-subcortical network that are specifically and differentially impacted by the type of antipsychotic treatment. Additional studies are needed to separate the contributions of clozapine-mediated neuroprotection, neurotoxicity related to typical antipsychotics, and the illness itself to the observed differences.

